# Electrochemical synthesis of Cu-pyrene MOF and its outstanding pohotoelectrocatalytic activity in hydrogen evolution reaction

**DOI:** 10.1016/j.isci.2026.116504

**Published:** 2026-06-26

**Authors:** Aso Navaee, Abdollah Salimi, Roushan Khoshnavazi, Keivan Akhtari

**Affiliations:** 1 Department of Chemistry, University of Kurdistan, Sanandaj 66177-15175, Iran; 2 Research Centre for Nanotechnology, University of Kurdistan, Sanandaj 66177-15175, Iran; 3 Department of Physics, University of Kurdistan, P.O.Box 416, Sanandaj, Iran

**Keywords:** Catalysis, Electrochemistry, Chemical reactions in materials science, Nanomaterials

## Abstract

A new procedure based on bipolar electrochemistry (BPE) is proposed for the synthesis of a copper-pyrene framework (Cu-py MOF). Accordingly, BPE using Cu mesh as BP electrode between two stainless steel driving electrodes is performed for 150 min under an applied potential of 3.0 V in acetonitrile containing 5% of 0.1 M NaOH aqueous solution. After characterization, the resulting compound is utilized for HER in water at electrolyte-free condition. Interestingly, the photocatalytic water reduction at the resulting material starts at 0.64 V relative to RHE, and photocurrent is increased with sweeping of potential in the cathodic direction, where the current density at an applied potential of 0.3 V vs. RHE reaches ∼30 μA cm^-2^, and a faradic efficiency of 1.01 μM cm^-2^h^-1^ of H_2_ is obtained. This proposed method has a great potential in the synthesis of organic polymers orincorporation of organic-inorganic materials to derive various organic semiconductors, MOF, etc.

## Highlights

• The Cu-pyrene MOF was synthesized via a simple and reliable electrochemical method
• The resulting MOF was utilized for photocatalytic HER at low over-potentials in water
• This MOF displayed much higher activity compared to the resulting MOF from chemical methods
• This can be a practical method for the sustainable synthesis of various MOF derivatives

## Graphical abstract

**Figure:**
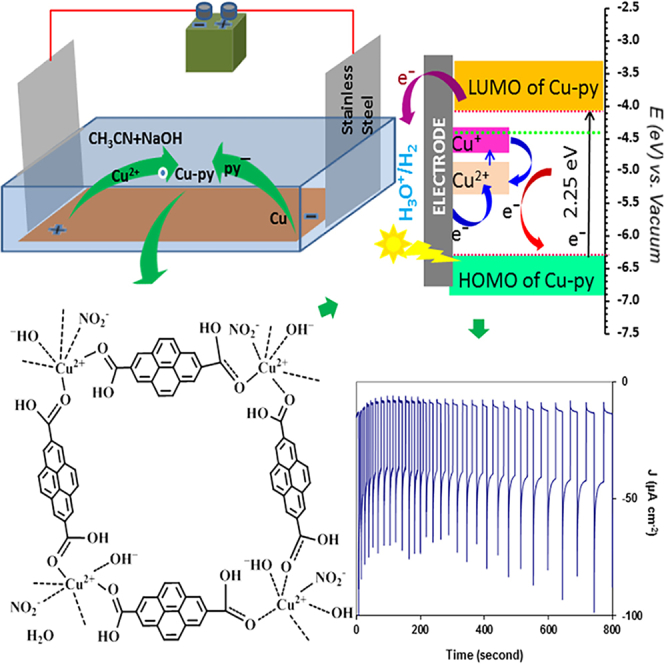

## Introduction

Organic semiconductors have shown great potential as an alternative to the traditional inorganic semiconductors.^1^^,^^2^^,^^3^ They have been utilized in many technologies such as catalysis,^4^^,^^5^ dye-sensitized solar cells,^6^^,^^7^ chemical sensors,^8^ organic field effect transistors,^9^ organic light emission diodes,^10^ and so forth. They have displayed exceptional charge carrier mobility,^2^^,^^11^^,^^12^ which strangely depends on the π-conjugation and HOMO/LUMO levels of organic molecules^13^^,^^14^^,^^15^ and make them proper for optoelectronic^16^ devices. One of the interesting applications of semiconductors is their catalytic ability in the energy conversion process toward the construction of green energy sources. Among many strategic processes, the critical one is catalytic water splitting to achieve H_2_ as a green energy source. However, from the thermodynamic viewpoint, this process is endothermic and requires total input energy of ΔH°=285.8 kJ mol^−1^.^16^ Therefore, photo-assisted conversions have attracted the highest attention in the recent decade, where significantly decrease the required input energy.^17^^,^^18^^,^^19^

Among numerous organic molecules to develop semiconductors, pyrene, a polycyclic aromatic molecule with extended π-conjugation, is known for its exceptional optical and electronic characteristics and therefore attracted much attention in optoelectronics and catalysis.^1^^,^^20^^,^^21^^,^^22^ The sp^2^ hybridization of whole carbon atoms in the pyrene unit leads to a planar structure with full resonance and electron delocalization, effectively enhances the interlayer π-π interaction and migration of carriers. Accordingly, such outstanding photoelectrical properties make pyrene derivatives as an attractive precursors for the synthesis of macromolecules and nanomaterials. To address these issues, they have been utilized as one of the basic building blocks for different organic architectures^1^ such as covalent organic frameworks (COFs)^20^ (for oxygen evolution reaction,^21^ HER;^23^ H_2_O_2_ production^24^ and lithium ion batteries^25^) as well as hydrogen-bonded organic framework (HOF).^26^ However, the photocatalytic activities of organic semiconductors have not been considered alike inorganic materials. Therefore, the one way to benefit from both organic and inorganic materials is the combination of both categories through surface hybridization of metals by organic species,^27^ or in the form of MOFs.^28^^,^^29^^,^^30^^,^^31^ Among them, MOFs as coordination polymers/networks have been developed as promising candidates due to their tunable structures, high surface areas, and diverse metal nodes and organic linkers. It has been observed that charge transfer from some metals (with low work function) to organic molecules at metal interfaces leads to the formation of polaron-like states and alters the electronic structure.^32^ So, through incorporation of inorganic centers with organic ligands, especially for those have extended π-conjugation, the electronic modulation and enhancement in photoelectronic and catalytic activities are expected,^33^^,^^34^^,^^35^ as can be naturally observed in enzyme and photosynthesis phenomena. The important key factor in increasing photoactivity assisted by metal/organic in the MOF structures is the fermi level position of metals and the HOMO/LUMO positions of organic linkers.^36^ In view of that, numerous applications of MOFs in photovoltaic, sensing, and catalysis area^37^ have been developed. Among those potential applications of MOFs, they have attracted much attention in the field of photocatalytic energy conversions.^31^^,^^38^ Recent studies have demonstrated that pyrenederivatives can be effectively utilized for the preparation of MOFs^28^^,^^29^^,^^39^ with improved photocatalytic performance.

On the other hand, among different metals, which act as metal centers in MOF structures, Cu has an interesting electronic configuration, which leads to outstanding chemical properties, such as catalytic and photocatalytic activities as well as a cofactor in different enzymes. Additionally, the non-toxicity and cost-effectiveness make Cu a prominent candidate for catalysts production, where Cu is the strongest catalyst in CO_2_ reduction reactions.^40^^,^^41^^,^^42^ With respect to the importance of copper and pyrene, the incorporation of them in the form of MOF can offer unique electronic properties and facilitate charge transfer processes in catalytic phenomena. Among various pyrene derivatives, 1,3,6,8-tetraphosphonate derivative^28^^,^^39^^,^^43^ or other complex substituted,^28^^,^^38^ have been widely used for MOF synthesis toward photocatalysis,^29^ such as photocatalytic HER^43^ and targeted chemodynamic therapy.^38^

Many synthesis procedures have been proposed to achieve MOF structures, which many of them are carried out through solvothermal conditions. Based on our knowledge, there are no electrochemical methods for the synthesis of pyrene-based MOFs. The bipolar electrochemistry (BPE) procedure, as a powerful, simple, and low-cost technique^44^ have been previously developed for the synthesis of molybdenum sulfide,^45^ polythiophene@Au NPs^46^ and graphdyine.^4^ In this research, BPE is performed as a rapid procedure compared to traditional hydro/solvothermal methods to prepare Cu-py MOF. After that, the photoactivity of the prepared MOF toward HER is evaluated. The outstanding photocatalytic activity without requiring any electrolyte is one of the interesting point in this research. Even though electrolyte supports the charge transfer between cathode and anode electrodes, it can leads to many complications, such as the cost of operation, polarization losses in the three-layer structure of cathode/electrolyte/anode compared to one layer in the electrolyte-free systems,^47^ precipitation on the cell and catalyst, toxic waste, and so forth. So, the electrolyte-free reduces many difficulties, as has been seen in solid oxide fuel cells.^47^^,^^48^ In such galvanic electrochemical systems, the utilized catalyst acts as both electrode and electrolyte.^49^ Morever, the electrolyte-free condition has been widely performed in organic synthesis.^50^^,^^51^^,^^52^

## Results

### Characterization of Cu-pyrene-based-MOF

Practically, most of the MOFs have been prepared using well-known chemical methods under thermal or mechanical conditions. Here, a simple and green electrochemical route based on the BPE is proposed, as schematically shown in Figure S1A. In this synthesis procedure, the affecting parameters such as medium of electrochemical synthesis, applied potential, distance of BP electrode to the driving electrodes, and time of electrochemical synthesis were optimized. Accordingly, at applied potentials above 5.0 V, the further corrosion of Cu mesh was seen. Whereas, at applied potentials below 2.0 V, the time of synthesis was increased, and also corresponding photocatalytic activity in both potential ranges above 5.0 V and below 3.0 V was low. The electrochemical procedure was also done without Cu BP electrode, but there was no photocatalytic activity for the resulting material. Overall, based on photocatalytic activity, the bias potential of about 3 V for around 150 min in acetonitrile containing 5% of 0.1 M NaOH aqueous solution is the optimized condition. As a result, the yellow color of the pristine pyrene solution (Figure S1B) changed to brown at the end of the BPE procedure. After that, the resulting mixture was diluted with water, and the dark yellow solution slightly changed to a green-brown chelate (Figure S1C), probably because of the accumulation of MOF fragments through hydrogen bonding or π-π stacking.

**Figure 1:**
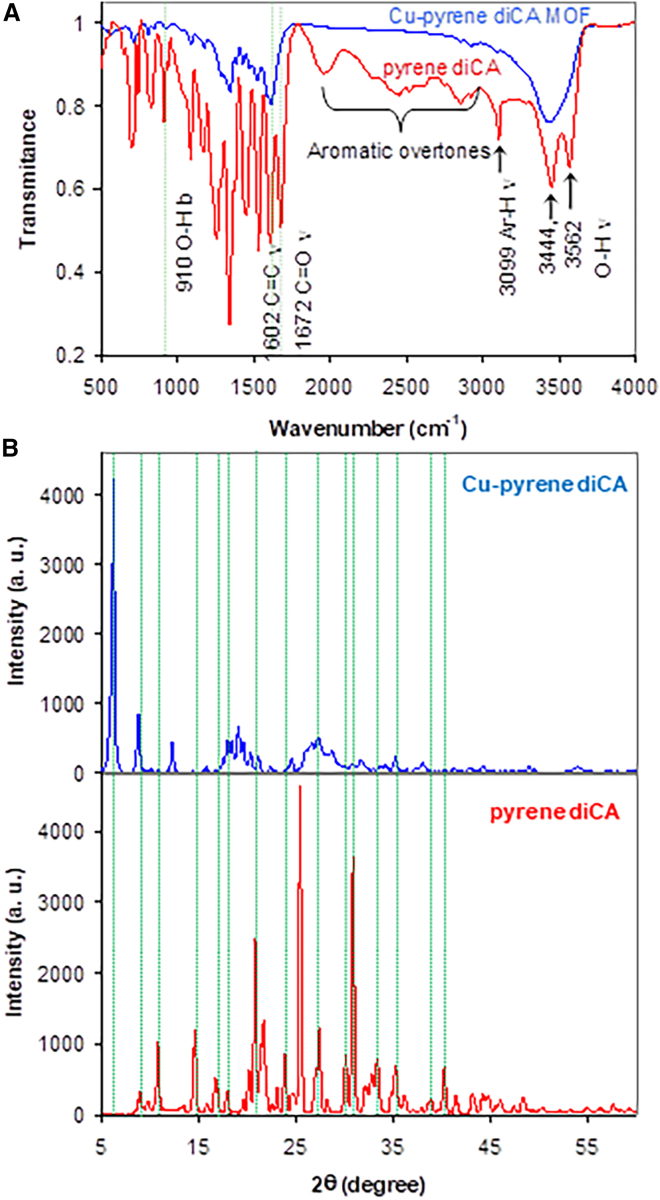
FT-IR and XRDcharacterizations of samples FT-IR spectra (A) and XRD pattern (B) of pristine pyrene dicarboxylic acid (pyrene diCA) and resulted Cu-py MOF.

To save time and cost of analysis, only the as-prepared material under optimized conditions was characterized by several techniques. Figure 1A represents the FTIR spectra of pristine pyrene as well as polymerized pyrene in the presence of Cu PB electrode. There are several noticeable changes in the FT-IR spectra. Outstandingly, pyrene shows an intense peak at 1672 cm^−1^ related to C=O stretching vibration of carboxylic acid functional group, while it disappears for the case of Cu-py, indicating the complexation of Cu cations with pyrene through HO-C=O and formation of Cu-O:::C. Another important difference is the disappearance of aromatic overtones around 1800–1970 cm^−1^ after Cu-py complexation, which may be because of intermolecular interaction^53^^,^^54^ after MOF formation. In the case of pyrene, there are doublet O-H stretching peaks at 3444 and 3562 cm^-1^, may be due to intermolecular hydrogen bonding between two neighboring carboxylic acids,^55^ whereas, in the case of Cu-py, it changes to a single broad peak, indicating the disruption of those hydrogen bond and coordination of Cu by those carboxylic acids. Moreover, the aforementioned O-H stretching vibration band for Cu-py at 3390 cm^−1^ can be related to Cu(O-H) stretching vibration.^56^ Another important change is the diminishing of the O-H bending peak at 910 cm^−1^ for the case of Cu-py. Therefore, the comparison of FT-IR spectra reveals the possible coordination of Cu by pyrene dicarboxylic acid, followed by polymerization through the electrochemical BPE procedure.

The possible crystalline phase of the prepared MOF was studied by XRD and compared with pristine pyrene dicarboxylic acid. As can be seen in Figure 1B, the diffraction patterns for both pristine and polymerized materials are complicated, which is expected for macromolecules or MOF structures as coordination polymer networks.^57^ On the other hand, to obtain a better understanding of the atomic arrangement of a crystalline material, single crystal XRD can provide a good description of that, but it requires a high-quality single crystal. Unfortunately, in most of the synthesis approaches, growing a single crystal MOF with enough quality is inaccessible, and usually, MOF powders are achieved.^57^ It has been suggested that strong X-ray absorption by conjugated polymer powders challenges on obtaining the appropriate signal -to -noise ratio.^58^ Moreover, the conjugated polymers mostly exhibit broad bonds^58^^,^^59^ because of low crystallinity. Here, the pristine pyrene dicarboxylic acid exhibits a complicated XRD pattern, where after MOF configuration, the diffraction peaks are completely differe from the pristine pyrene, in which a strong diffraction peak at 2θ of around 6.2° corresponds to the appearance of conjugated carbon materials. Additionally, other broad peaks at 2θ of 8–35°are seen, with the small and narrow peaks appearing on them, resemble the XRD spectra of the metal-organic complexes.^60^ In comparison, other diffraction peaks for the case of the resulting MOF have been shifted or diminished. Finally, there are no evidence peaks related to crystal configuration of CuO, Cu_2_O, suggesting the coordination of Cu^n+^ with pyrene, and may possibly, with counter ions. Because of the complexity in the diffraction peaks of the resulting MOF, a definite PDF card number has not been achieved.

**Figure 2:**
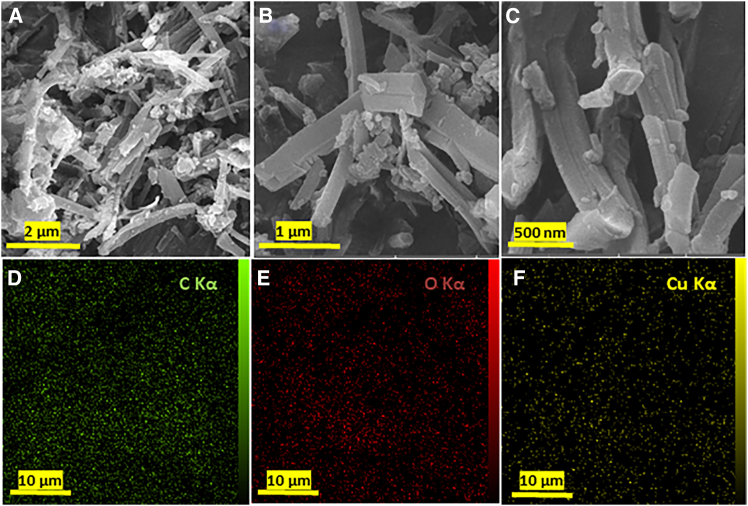
SEM and map analysis of prepared MOF SEM micrographs with different magnifications (scale bars, for A is 2 μm, for B is 1 μm, for C is 500 nm) and map micrographs (D, E, F with scale bars of 10 μm) of the resulting Cu-py.

**Figure 3:**
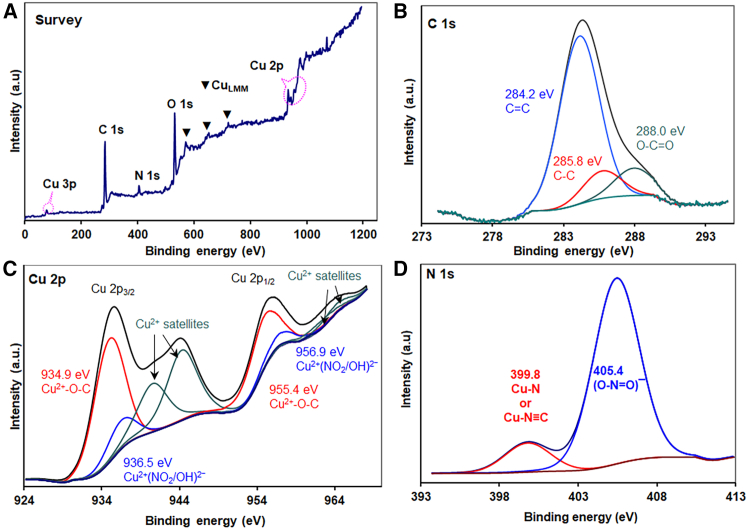
XPS analysis of prepared MOF Survey XPS spectra of the resulting Cu-py (A) as well as curve fitting of narrow scans of C (B), Cu (C), and N (D) elements.

SEM imaging is a powerful technique for evaluating the morphology of the micro/nanostructures. Figure 2 (a, b, (3) represents the SEM micrographs at different magnifications, which reveal the microscale morphology of the as-prepared compound through optimized synthesis conditions. The microstructures of the as-prepared material look like irregular square prisms that randomly orient and accumulate on each other with lengths of ∼0.5–4 μm and diameters of ∼200–400 μm, which supports the achievement of MOF structure. Higher SEM resolution reveals the tiny nanostructures as building blocks of the MOF microstructure (Figure S2). On the other hand, the wide area SEM micrograph has been presented in Figure S3A and corresponding map images are observed in Figure 2 (d, e. f), representing the well distribution of C, O, and Cu. It should be noted that a very small amount of Fe element is seen in the resulting MOF (Figure S3B), which has originated from stainless steel driving electrodes. The elemental analysis spectra using EDX for the resulting structure have been presented in Figure S4, and the corresponding atomic percentage of elements are shown in the inset of the same Figure, demonstrates 54% of C, 2.5% of Cu, and a small amount of Fe. Therefore, the ratio of pyrene to Cu is obtained to be ∼2:1.

The porosity and specific surface area of the resulting material were estimated by Brunauer-Emmett-Teller (BET) analysis based on the N_2_ adsorption isotherm at 77 K. As shown in Figure S5, the adsorption/desorption isotherm of the resulting material displays a Type-H3 hysteresis loop according to the IUPAC classification, where the adsorption branch resembles the type II isotherm. This type of isotherm is seen in non-rigid aggregates of plate-like particles.^61^ The specific surface area of 4.04 m^2^ g^−1^, total pore volume of 0.004 cm^3^ g^−1^ and pore diameters of 4.18 nm were found, signifying micro-pores with low pore volume and diameter. The narrow channels and low specific surface area can be related to the π-π stacking of conjugated pyrene, which tightly aggregates to each other and restricts N_2_ transfer into the micro-pores.

**Figure 4:**
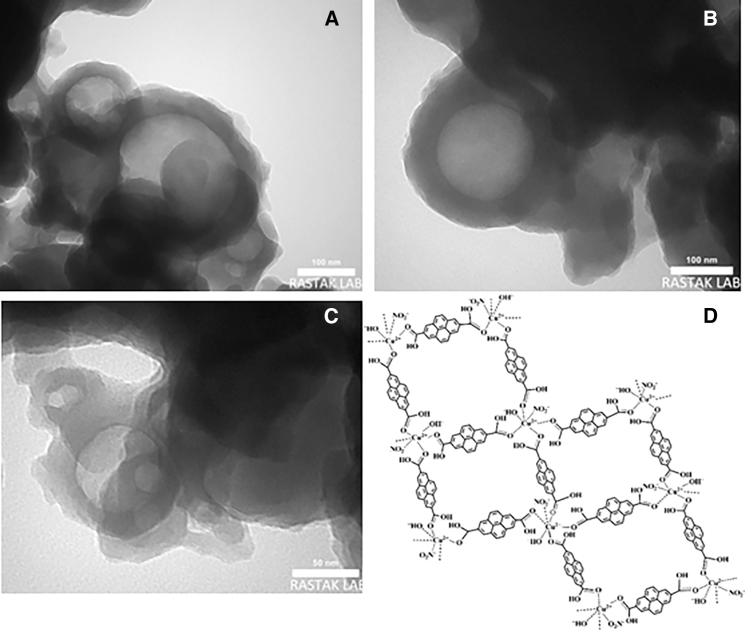
TEM images of the resulting Cu-py MOF at nanoscale magnification The scale bars for the cases of A and B, are 100 nanometers and for C are 50 nanometers. (D) The proposed chemical structure is based on different characterization tools as discussed above.

To reveal the atomic composition and nature of bonds between atoms in the resulting structure, as well as their oxidation states, XPS analysis was performed. The survey spectrum (Figure 3A) reveals the existence of 65.3% C, 26.6% O, 5.8% N ,and 2.2% Cu, in atomic percentage, and there are no other elements in that compound (Figure 3). Therefore, the ratio of C to Cu is about 30:1, indicating ∼2:1 pyrene to Cu. Moreover, the ratio of Cu to N is about 1:1.4. Based on these values and TEM images, the resulting MOF can have the proposed structure that has been presented in Figure 4. However, slight differences in atomic composition obtained by EDX rather than XPS analysis are seen, which can be attributed to the differences in the X-ray penetration depth in these two techniques, where EDX provides more information from the bulk of the materials rather than the XPS technique. On the other hand, the vacuum level in these two techniques can make a difference in O_2_ fraction, and overall atomic percentages. The narrow scan of C, N, O, and Cu was fitted to reveal the electronic structure of each element (Figure 3; Figure S6). The deconvoluted peaks at 284.2, 285.8, and 288.0 eV signify C=C, C-C, and O=C-O, respectively (Figure 3B). In the case of oxygen (Figure S6), there are three XPS peaks at 531.1, 532.7, and 534.3 eV, attributed to oxygen in contact with Cu^n+^, C, and N, respectively. The fitting of the Cu 2p peak (Figure 3C) reveals intense satellite peaks that approve existing of Cu^2+^. Compared to the literature,^62^ the peaks of Cu have slightly shifted to the higher binding energies, which indicates the coordination of Cu with π-acceptor ligands.^63^ Deconvolution of Cu 2p_3/2_ reveals two peaks at 934.9 eV and 936.5 eV, representing Cu-O and Cu-NO_2_, respectively. The related Cu 2p_1/2_ can be seen at 955.4 eV and 956.9 eV, respectively. Similarly, coordinated structures of copper with nitrite ion are naturally produced during nitrogen oxide processing by nitrite reductase enzyme,^64^ and artificial models have been chemically synthesized.^65^^,^^66^ The resulting Cu-nitrite is more confirmed by the fitting of the narrow scan of nitrogen. Remarkably, the result compound contains 5.8% of N, which curve fitting indicates that the most intense peak of N is located at 405.4 eV, proving the nitrite ion, originated from oxidation of acetonitrile through BPE process (Figure 3D). Therefore, through electro-generation of Cu^n+^ cations from BP Cu metal, the NO_2_^−^ counter anions are derived from oxidation of acetonitrile solvent, which alongside OH^−^, leads to compensation of the charge neutrality and achieving charge balance at the resulting MOF structure. Therefore, alongside each Cu^n+^, n NO_2_^−^/OH^−^ can exist, and Cu centers are coordinated by carboxylic acid functional groups of the pyrene ligand as well as NO_2_^−^/OH^−^. As it will be discussed later, counter ions as the host enhance the adsorbtion of polar solvents and polar species such as H_3_O^+^, facilitate the charge transfer at the interface of MOF/electrode. Another weak peak of nitrogen at 399.8 eV signifies nitride or cyanide (Cu-N or C≡N-Cu). Overall, XPS results prove the proposed structure in Figure 4.

To obtain the structural information at the nanoscale dimension, the resulting structure was evaluated by transmission electron microscopy (TEM), which provides 2D images with higher resolution rather than 3D images of SEM micrographs. As can be seen in Figures 4A–4C, in the condensed areas, the resulting material looks like hollow shapes. However, in the frailer portions with higher magnification, ring-like planar structures are observed, which polymerization of Cu-py complex units has led to the formation of these ring-like walls followed by hollow structures through growthing (Figure 4 and S7). Then, the MOF microstructure is formed through the arrangement of these hollow shapes. Therefore, based on the presented characterizations and the ratio of pyrene to Cu, the chemical structure of the resulting MOF is expected to be the proposed structure shown in Figure 4D.

### Optical and electrochemical characteristics of the resulting Cu-py

**Figure 5:**
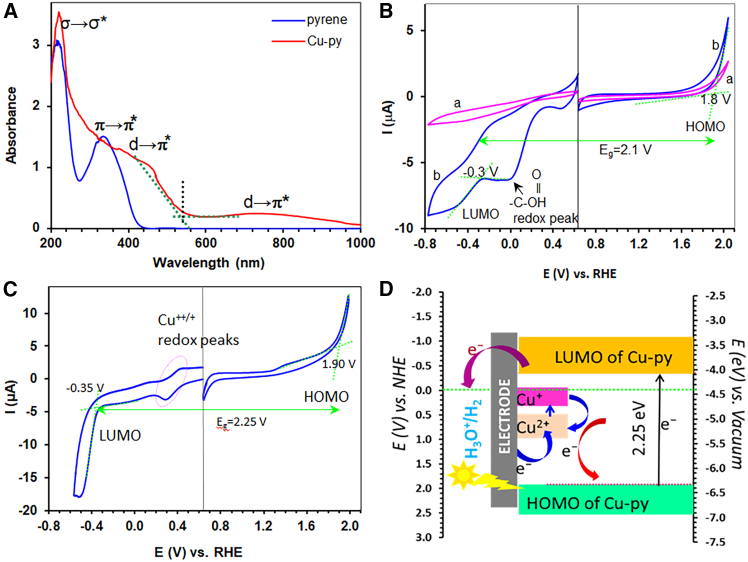
Optical and electrochemical properties of samples (A) Uv-Vis spectra of pristine pyrene dicarboxylic acid and Cu-py MOF. (B) CV of bare GC (A) and 0.1 mM pyrene dicarboxylic acid (B) in water in both cathodic (left) and anodic (right) directions. (C) CV of Cu-pyrene MOF in water in cathodic (left) and anodic (right) directions. (D) The calculated band edges position and band gaps as well as the proposed mechanism of electron transfer.

One of the important characteristics of conjugated materials is the charge transfer induction under light irradiation. The optical properties of Cu-py MOF were studied by Uv-Vis spectroscopy to evaluate the energy gap between the highest occupied molecular orbital (HOMO) and the lowest unoccupied molecular orbital (LUMO), which in semiconductors are called the valence band and the conduction band, respectively. Figure 5A exhibits the Uv-Vis spectra of pristine pyrene and as-prepared Cu-py. Both components have displayed an absorption band at around 220 nm, which can be attributed to the n→σ∗ transition, and another band around 350 nm, attributed to π→π∗ transition. However, the second peak in Cu-py has been extended from 300 to 550 nm, signifying both π→π∗ and d→π∗ contributions in conjugated Cu-py. Moreover, in the case of Cu-py, a broad peak with weak intensity (whose middle is located at ∼720 nm) is also seen, assigning another d→π∗ transition, confirming the formation of a conjugated metal organic framework. Regarding the offset lambda of intense transition (*λ*_*edge*_=550 nm for Cu-py) and photon energy formula, *E*_*g*_*=1240/λ*_*edge*_, the optical amount of energy gap between HOMO and LUMO (*E*_*g*_) can be approximated^4^^,^^67^^,^^68^ around 2.25 eV for Cu-py and 2.75 eV for pristine pyrene.

On the other hand, *E*_*g*_ as well as positions of HOMO (*E*_*HOMO*_) or valence band and LUMO (*E*_*LUMO*_) or conduction band can be approximated by cyclic voltammetry through scanning of the working electrode modified by the understudied substance in its oxidation and reduction window (Figures 5B and 5B). Also, in the absence of pyrene, the bare GC electrode at both cathodic and anodic directions was scanned for comparison, where two smooth CVs are seen (Figures 5B and 5A). Through anodic sweeping, the onset oxidation potential (E′_ox_) for pristine pyrene solution and Cu-py (coated on GC electrode) appear at ∼1.80 and ∼1.90 V, respectively. In the cathodic direction, for the case of pyrene, a cathodic peak related to the carboxylic acid functional group appears at around 0.0 V (Figures 5B and 5B), while this peak disappears for the case of Cu-py and instead of that, a redox peak related to Cu^2+^/Cu^+^ appears (Figure 5C). With swiping potential in a negative direction, the onset reduction potentials (E′_red_) related to pristine pyrene and Cu-py appear at ∼ -0.30 V and ∼-0.35 V vs. RHE, respectively. Therefore, the E_g_ values for pyrene and Cu-py are estimated about 2.10 and 2.25 eV, respectively. Subsequently, *E*_*HOMO*_ and *E*_*LUMO*_ vs. vacuum levels could be calculated according to the following equations^69^

(Equation 1) $$E_{HOMO}=-I_{p}=-\left(E_{OX\;\left(vs.\;NHE\right)}^{\prime }+E_{e}\right)eV$$

(Equation 2) $$E_{LUMO}=-E_{a}=-\left(E_{red\;\left(vs.\;NHE\right)}^{\prime }+E_{e}\right)eV$$

where I_p_ and E_a_ are the ionization potential and electron affinity, respectively. E_e_ is the energy of a free electron in the vacuum, equal to ∼4.45 eV.^70^ Consequently, E_HOMO_ and E_LUMO_ for Cu-py have been estimated as −6.35 eV and −4.10 eV, respectively. Therefore, the amount of E_g_ calculated by cyclic voltammetry is about 2.25 eV, almost similar to the optical value. Whereas, those values for pyrene are slightly lower than Cu-py and E_g(CV)_ differs from E_g(OPT)_. It has been seen that sometimes estimation of LUMO energy by the optical method may differ from the actual level.^71^^,^^72^ However, the amount of E_g_ corresponds to the energy of visible light, and electron transfer from HOMO to LUMO can occur under visible light irradiation. On the other hand, the LUMO position energy (−0.3 V vs. RHE) is appropriate for HER (0.0 V vs. NHE/RHE at pH=0, −4.45 eV vs. vacuum). The overall band edge positions and proposed electron transfer mechanism are shown in Figure 5D. It is suggested that Cu^2+^ centers receive electrons from the electrode and facilitate transfer to the π-conjugated Cu-pyrene network through Cu^2+^/Cu^+^ redox reaction. Thus, through light irradiation, electrons are transferred to the LUMO state, which is located at a higher energy than the H_2_O reduction state.

### Computational modeling

**Figure 6:**
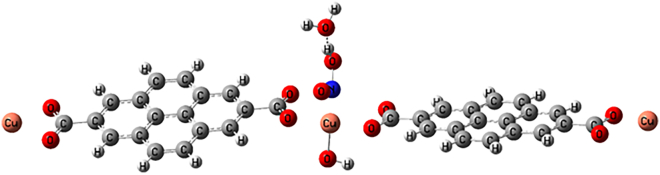
The optimized geometry of the complex of a hydronium and Cu(I)-py

**Figure 7:**
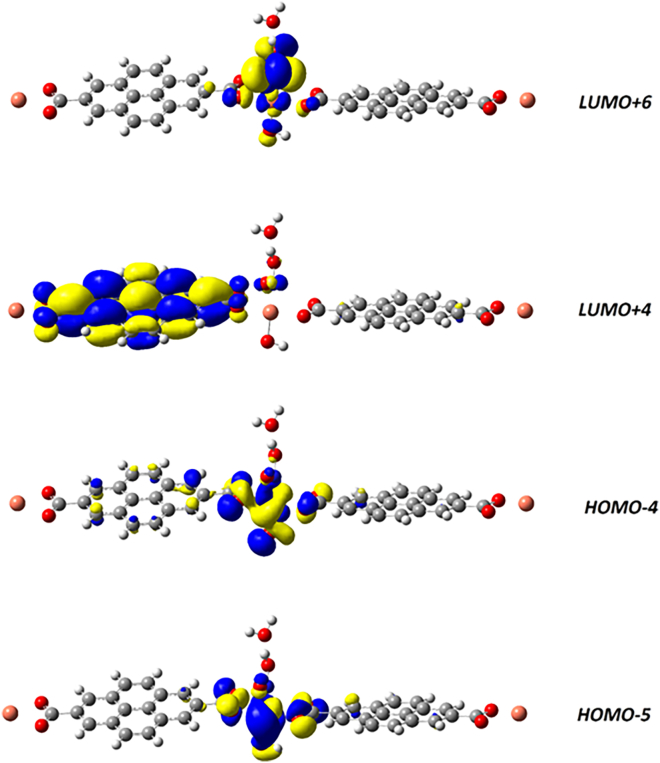
The plot of contributing orbitals involved in the main transitions as mentioned in Table 1, obtained at the PBE0/[6–31G(d,p) + Lanl2dz (Cu)] level of theory, for the investigated system (HOMO-4 and HOMO-5 refer to the fifth and sixth highest occupied molecular orbitals, respectively, while LUMO+4 stands for the fifth lowest unoccupied molecular orbital)

Based on the standard reduction potentials, Cu^2+^→Cu^+^ occurs at 0.35 V vs. RHE, and applying 0.30 V in this research is sufficient to induce this process. Therefore, in order to simulate electron transition under photoelectrocatalytic reaction of Cu-py structure, the last oxidation state of Cu has been considered, and a small model of Cu(I)-py/NO_2_^−^/H_3_O^+^ has been designed, and density functional theory (DFT) calculation was performed based on the available program, which has been provided in Method S1 in the supplementary materials. Figure 6 shows the optimized geometry of the model. The photo-excitation parameters for strong transitions have been presented in Table 1. As can be seen, all transitions have the d→π∗ character. Figure 7 shows the molecular orbitals that have a major contribution to charge transitions. The largest contributions (except HOMO-5→LUMO+(6) involve the electron transition from the d orbital of the central copper atom to π∗ of the protonated site of the reaction center. The DFT-based calculations explain the enhanced photocatalytic behavior of the Cu-py in its reduced form.

**Table 1:** Calculated parameters obtained from TD-DFT calculations for the model among 18 transitions

Number of transitions	*λ*_*max*_	*E*_*max*_	*f*_*os*_	Major contribution	Character of transitions
3	804	1.54	0.042	HOMO →LUMO+6 (73%)	d →π∗
4	743	1.67	0.038	HOMO →LUMO+4 (79%)	d →π∗
13	426	2.91	0.361	HOMO-4→LUMO+6 (44%) HOMO-5→LUMO+6 (21%)	d →π∗ d →π∗

The above characteristics are: wavelength (*λ*_*max*_), oscillator strengths (*f*_*os*_ ≥ 0.01), transition energies *E*_*ma*_ (eV), and major molecular orbital contribution to the intramolecular charge-transfer and their characters.

### Photoelectrocatalytic activity toward HER

**Figure 8:**
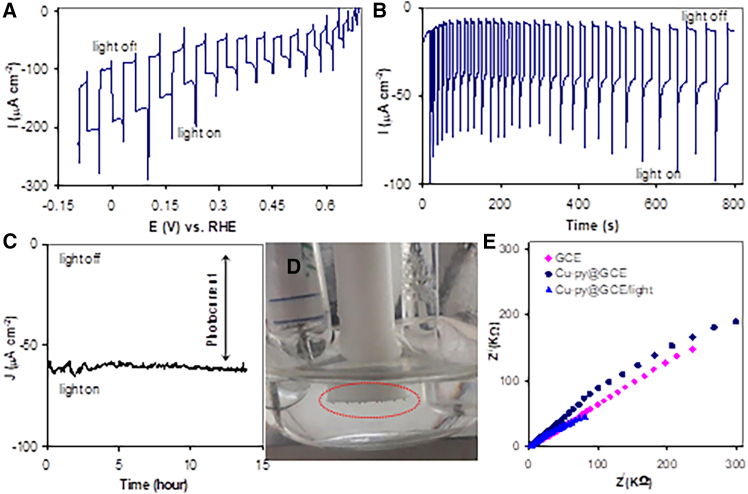
Recorded LSV by Cu-py thi n film coated on GCE without and under light irradiation (A) and its chronoamperometry curve to investigate photoactivity under light on/off at a potential of 0.3 V vs. RHE (B) in DI water (C) Time stability of photoactivity recorded by chronoamperometry under light irradiation at a potential of 0.3 V vs. RHE. (D) The corresponding actual H_2_ bubbles on the electrode surface. (E) Electrochemical impedance spectra of bare GCE, Cu-py on GCE before (circle dots) and after light irradiation (three angle dots) at a potential of 0.3 V vs. RHE and a frequency range of 100 kHz to 0.1 Hz.

To investigate the photocatalytic activity of the resulting Cu-py MOF at various applied potentials, linear sweep voltammetry (LSV) was performed under or without light irradiation at potential ranges of 0.7 V to -0.1 V vs. RHE. As can be seen in Figure 8A, through light irradiation, the current intensity is simultaneously increased with the increase in overpotential. In potential ranges of 0.7 to 0.3 V, the simultaneous increase in photocurrent is greater than in potential ranges of 0.3 to -0.1 V. However, these results depict the dependence of output photocurrent on applied potentials. To reveal the effect of light irradiation on the response of the catalyst at a constant potential, the potential of -0.3 V vs. SCE (0.3 V vs. RHE) was selected as a middle potential for the chronoamperometry experiment (Figure 8B). Under light illumination, current is rapidly increased as much as ∼54 μA cm^−2^, while, after lighting off, current is rapidly diminished. These phenomena represent the fast electron transfer and photocatalytic activity of the catalyst. This behavior repeats for several cycles, and there are no decreases in the resulting photocurrent. The long-time stability of the catalyst is also evaluated for ∼14 h, and a good stability is observed (Figure 8C). The theoretical amount of released H_2_ is calculated according to Equations S1 and S2 in the Method S2 section, and obtained as much as ∼1.01 μM cm^−2^ h^−1^ H_2_ (∼30.1 μL cm^−2^ h^−1^). The released bubbles of H_2_ as the nonpolar molecule are gathered on the Teflon cover of the electrode, as the actual image are seen in Figure 8D and S8. These results show the potential application of the resulting MOF for hydrogen generation from electrolyte-free water in the natural pH.

To compare the performance of the electrochemical synthesis procedure with chemical methods, Cu^2+^ was interacted with pyrene dicarboxylic acid in ACN solvent containing 5% of 0.1 M NaOH under three conditions: a) room temperature for 7 h and b,c) solvothermal conditions at 150 °C for 7 and 22 h, respectively. The Uv-Vis spectra for pyrene and all resulting materials have been presented in Figure S9A. As can be seen, the resulting material from electrochemical synthesis displays a broader spectrum, which, according to the offset λ_edge_, has a lower band gap. For the cases of room temperature and solvothermal conditions for 7 h, almost a similar offset λ_edge_ is observed, while the resulting material under 22 h conditions has a lower offset λ_edge_ and a higher band gape than others. Remarkably, a and b display a similar photocatalytic activity, and it decreased for the case of c, as can be observed in Figure S9B. Consequently, the material resulting from the electrochemical procedure displays ∼2 times higher activity than the materials resulting under conditions of a and b, and ∼2.5 times higher activity than the materials resulting from condition of c. Therefore, the proposed electrochemical synthesis (BPE) is a more satisfactory technique than chemical methods.

It should be noted that a small amount of Fe was detected by EDX analysis (Figure S4), but it had no evidence impact on photoactivity. It was confirmed when the Cu BP electrode was replaced by a Fe BP electrode and the resulting material did not show any evidence photoactivity. Therefore, as explained above, Cu^2+^ has a decisive impact on the HER phenomenon. Moreover, the effect of pyrene was also confirmed by utilizing copper oxide synthesized through our previous work,^73^ and there was a negligible photocatalytic activity under the present experimental conditions (Figure S9b), proving the synergistic effect of Cu and pyrene in HER application, as schematically shown in Figure 5D.

One of the interesting issues of this work is the occurrence of HER in the pure water free of electrolyte, whichpresence of electrolytes, sometimes causes some drawbacks. Basically, water molecules surround the Cu-py at the interfaces of the electrode because of the existence of NO_2_^−^ and OH^−^ counter-ions presented in the Cu-π-conjugated pyrene backbone (the proposed structure given in Figure 4D). This structure can act as both electrolyte and adsorbent for H_3_O^+^, as has been previously investigated that counter-ions at host compounds lead to adsorption of small gases and solvent molecules.^74^^,^^75^ For instance, OH^−^ treatment of MXene has improved the adsorption of water.^76^ On the other hand, enhancement in HER has been seen from bifunctional MOFs.^77^ Additionally, swiping potential in a negative direction induces adsorption of more water molecules at the interface through formation of an extended interfacial layer of H-bonded water, as has been seen for the Pt electrode in contact with ionic liquid.^78^ Consequently, those impacts enhance the accessibility of water for postreaction at the electrode interface.

The effect of light on the resistance of the coated catalyst on GCE was studied by electrochemical impedance spectroscopy (Figure 8E). Compared to GCE, the Cu-py MOF leads to a slight increase in the total impedance of the electrode. Interestingly, under light illumination, the total impedance is drastically decreased, suggesting that light illumination induces interfacial electron transfer. With regard to the HOMO-LUMO position and E_g_ value, such incidents are expected. The overall results demonstrate the improvement of electron transfer at the resulting MOF under light illumination.

As mentioned above, an appropriate photocatalytic response of the catalyst with a current density of ∼54.1 μA cm^−2^ is observed, and there is almost a constant photocurrent through successive light switching. The incident photon to current efficiency (IPCE), which expresses the probable efficiency of hydrogen generation from water assisted by light, is calculated using the following equation:

$$IPCE=\frac{number\;of\;electron\;generated\;by\;light}{number\;of\;incident\;photons}=\frac{J\times E}{W\times e}=\frac{J\times \frac{hc}{\lambda }}{W\times e}$$

where *J* is the photocurrent density (A cm^-2^ equal to columbs cm^−2^ s), E is the photon energy, W is the irradiated light power at specific wavelength (watt per cm^−2^ equal to joule s cm^−2^), e is the charge of electron equals to 1.6 × 10^−19^ coulombs, c is the light speed (3 × 10^8^ m s^−1^), *h* is the Planck constant (6.626 × 10^−34^ joule s) and λ is the wavelength of light absorbed by photocatalyst in meter (∼400 × 10^−9^ m for Cu-py). Here, the IPCE% is calculated to be 2.50%. This value is comparable or higher than some of previously reported works (Table 2).

**Table 2:** Comparison of some photon to current efficiency in catalytic HER

Material	Method of synthesis	Applied potential	IPCE%	Reference
MoSx@dye-sensitizer	mechano-chemical	Cathodic bias of 0.0 vs. NHE at pH=0	1.54	Click et al.^79^
MoS_x_@poly-salicylic acid	BPE	Cathodic bias of 0.1 V vs. RHE at pH=0	0.62	Lotfi et al.^45^
TiO2 nanosheet	hydrothermal	Anodic bias of 0.5 V vs. Ag/AgCl pH=6.5	3.7	Qin et al.^80^
(Au NPs)/p-type NiO/platinum film	sputtering	Cathodic bias of -0.7 vs. Pt in 0.1 M KClO4	0.45	Oshikiri et al.^81^
TiO2-Al-Zn-Ce	sol-gel	Anodic bias of 0.8 V vs. Ag/AgCl; pH=6.5	0.28	Hao et al.^82^
Cu-pyrene MOF	BPE	0.3	2.50	This work

## Discussion

In this research, we present a comprehensive investigation of the synthesis, characterization and photoelectrocatalytic performance of the Cu-py MOF in HER. Accordingly, a very rapid, simple, and cost-effective electrochemical procedure has been proposed for the synthesis of Cu-py MOF, in which the Cu ions have been slightly generated during the bipolar procedure, and therefore they have enough time to react with pyrene dicarboxylic acid ligands for the formation of a highly distributed MOF structure. Besides pyrene, the Cu centers are also coordinated with nitrite and hydroxyl groups. Such coordination environment around the Cu centers, combined with the π-conjugated nature of pyrene, leads to enhanced adsorption of water and facilitates the desorption of generated hydrogen, thereby improving the overall reaction kinetics. The energy of the HOMO/LUMO position and mechanism of charge transfer were clarified using experimental and DFT calculations. As was seen, the as-synthesized Cu-py MOF leveraged the synergistic effects of Cu and pyrene in the conjugated Cu-py backbone for efficient photoelectrocatalytic HER at low bias potential (-0.3 V vs. SCE at pH=6.5, 0.3 V vs. RHE) in an electrolyte-free environment assisted by a very low light source. These outcomes promise a new insight into the synthesis of next-generation catalysts based on organic semiconductors and organic/inorganic coupled material in the form of MOF for sustainable energy conversion and other catalytic areas.

### Limitations of the study

This work proposed a new procedure for the synthesis of Cu-pyrene MOF. Although the procedure is simple, and the prepared material has shown outstanding photocatalytic activity in HER, both preparation and application have some limitations. The cost of pyrene dicarboxylic acid is a limitation factor for the synthesis of MOF- based catalysts compared to inorganic counterparts. Related to the synthesis procedure, the key factor is the control of the BP electrode distance from the driving electrodes, which should be carefully located near each other. Another problem is the purification of MOFs to obtain the single crystal, as mentioned in the XRD characterization section. Here, the co-precipitation of unreacted pyrene with the resulting Cu-pyrene MOF decreases the purity of the proposed catalyst. Related to the photocatalytic study, low reproducibility in casting the thin layer on the holder or current collector is a disadvantage, while this limitation is overcome by applying several repeated CV cycles.

## Resource availability

### Lead contact

Further information and other requests should be directed to and will be fulfilled by the lead contact, Aso Navaee (aso.navaee@uok.ac.ir).

### Materials availability

This work did not generate new unique reagents. All materials and methods used for data generation and analysis have been mentioned in the article.

### Data and code availability

All data reported in this article will be shared by the lead contact upon request. This article does not report original code. Any additional information required to analyze the data reported in this article is available from the lead contact upon request.

## Acknowledgments

Authors acknowledge the Research Office of the 10.13039/501100008973University of Kurdistan (grant number of 02/9/33564) and the Iranian National Science Foundation for supporting of this work.

## Author contributions

Data curation, formal analysis, writing, and review of the original draft were done by A.N. Supervision of the work is A.S. The electrochemical instruments were supplied by R.K.. The theoretical DFT modeling was done by K.A.

## Declaration of interests

The authors declare no conflict of interest.

## STAR★Methods

### Key resources table

**Table:** 

REAGENT or RESOURCES	SOURCE	IDENTIFIER
Pyrene-2,7-dicarboxylic acid	PubChem	22340556
Cu foam >99%	Redoxkala	Tehran, Iran
Acetonitrile	Merck	CAS 75–05–08
Sodium hydroxide	Merck	1310–73–2

**Software and algorithms**		

Gaussian 98 program	GAUSSSUM 2.2	https://gausssum.sourceforge.net
EXCEL	MICROSOFT OFFICE	https://www.office.com

**Other**		

UvVis spectra	SPECOL 250 from	Analytic Jena
FTIR spectra	VECTOR-22	BRUKER spectrophotometer
X-ray Diffraction (XRD)	GNR EXPLORER	Italy
Scanning Electron Microscopy (SEM) along with Map and Energy Dispersive X-ray (EDX)	MIRA3 TESCAN	CHZEK REPUBLIC
X-ray Photoelectron Spectroscopy (XPS)	VG Microtech	Twin Anode, XR3E2 X-ray source using Al Kα (hν=1486.6 eV)
Transmission Electron Microscopy (TEM)	Philips EM208S KB	Netherland
Brunauer-Emmett-Teller (BET)	MicrotraceBEL	Corop
Voltammetry and Amperometry	PGSTAT 101	AUTOLAB

### Experimental model and study participant details

Our study does not involve biological models.

### Method details

#### Electrochemical synthesis of Cu-py

The BPE is performed in a homemade cell using a desired Cu mesh as BP electrode according to the cell (here, 0.5 × 1.0 cm), between 2 Stainless Steels as driving electrodes. The BP electrode was inserted horizontally with distance of ∼1.0 mm between two vertically driving electrodes (the important task is the distances between driving electrodes and BP electrode that should be situated very close to each other). This procedure was performed in water, ethanol, pure acetonitrile (ACN) and 1:1 ethanol: acetonitrile. Based on the photocatalytic activities of resulted materials, we find that a solution of 0.1 M of pyrene dicarboxylic acid in acetonitrile containing 5% of 0.1 M NaOH aqueous solution is the best medium for synthesis. The BPE procedure was performed in different potentials (1.0–7.0 V) and times (30–300 min) to optimize the synthesis conditions. Accordingly, potential of 3.0 V for 150 min until the yellow color of solution changed to green brown was find as the optimize condition. The resulted compound was diluted by water, and then centrifuged at 3000 rpm to achieve the precipitate.

#### Chemical synthesis of Cu-py

In order of comparison the electrochemical (BPE) with chemical procedure, three solutions were prepared as follow: 1 mmole of Cu(NO_3_)_2_.3H_2_O was dissolved in around 20 ml of ACN and after that 1 mmole of pyrene dicarboxylic acid was added to that solution under stirring. After complete dissolving, about 1.0 ml of NaOH 0.1 M was added dropwise to that. After that, one of those solutions was stirred at room temperature for 7 h. The two other solutions were transferred to the Teflon-lined Autoclave and putted at 150°C for 7 and 22 h, respectively. Finally, the resulted supernatant was washed with ACN, ethanol and water under centrifugation.

#### Materials characterization

UvVis spectra were recorded by SPECOL 250 from Analytic Jena, FTIR spectra with VECTOR-22 BRUKER spectrophotometer, X-ray diffraction (XRD) spectra with GNR EXPLORER and scanning electron microscopy (SEM) micrographs along with Map and energy dispersive X-ray (EDX) analysis were taken with MIRA3 TESCAN. X-ray photoelectron spectroscopy (XPS) analysis was recorded by a VG Microtech Twin anode XR3E2 X-ray source and operated at 5 × 10^−10^ mbar using Al Kα (hν=1486.6 eV). Binding energies were referred to C 1s peak at 284.5 eV. Transmission electron microscopy (TEM) images were taken by Philips EM208S KB, from the Netherland. Brunauer-Emmett-Teller (BET) was taken by MicrotraceBEL Corop. Voltammetry and amperometry measurements were recorded by Autolab PGSTAT 101 equipped with three-electrode system, an Ag/AgCl, Pt wire and glassy carbon electrode (GCE) as reference, counter and working electrode, respectively.

### Quantification and statistical analysis

There are no quantification or statistical analyses to include in this study.

## Supplemental information

Document S1. Figures S1–S9, Methods S1 and S2, Supplemental references
