# Supplementary material for: Electrochemical synthesis of Cu-pyrene MOF and its outstanding pohotoelectrocatalytic activity in hydrogen evolution reaction
Source: iScience. 2026 Jun 26;29(7):116504. doi: 10.1016/j.isci.2026.116504 (PMC13378021; doi:10.1016/j.isci.2026.116504)
Supplement: Document S1. Figures S1–S9, Methods S1 and S2, Supplemental references [file mmc1.pdf]

iScience, Volume *29*

Supplemental information

Electrochemical synthesis of Cu-pyrene MOF and its outstanding pohotoelectrocatalytic activity in hydrogen evolution reaction

Aso Navaee, Abdollah Salimi, Roushan Khoshnavazi, and Keivan Akhtari

METHOD S1

Computational modeling program

In order to simulate electron transition of the Cu(I)-Py/H_3O^+ structure, a small model has been designed. Geometry optimization carried out, using density functional theory method. The PBE0 functional, the standard 6-31G(d,p) for light atoms and the LanL2DZ basis sets for Copper atoms were used. The Time-Dependent Density Functional Theory (TD-DFT) was used to calculate the excited state energies. The 18 lowest excited states have been considered. All calculations were performed using Gaussian 98 program [1]. To estimate the solvent effect, we performed polarized continuum model (PCM) method calculations [2]. The orbital transition contributions were obtained using GAUSSSUM 2.2 program [3].

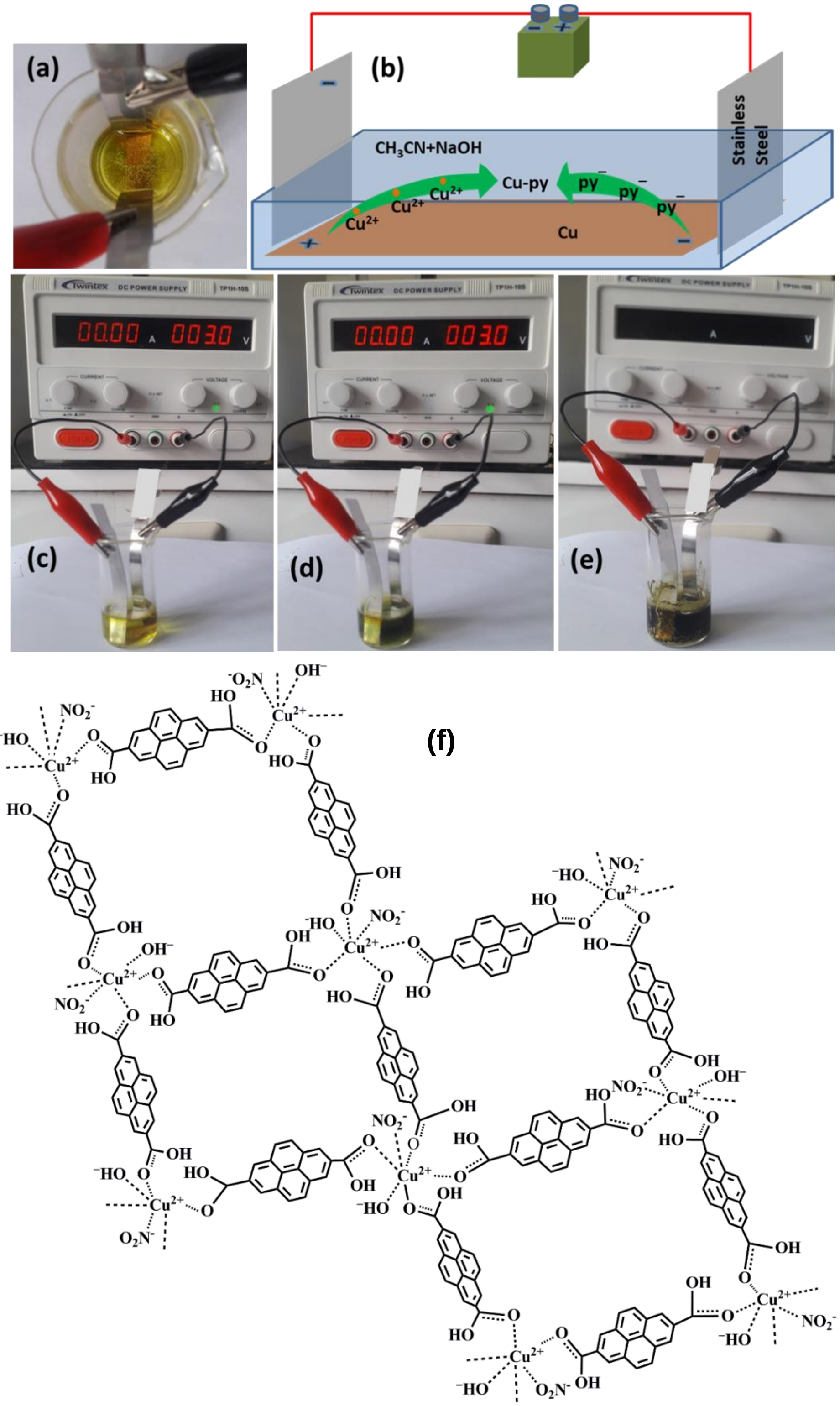

Figure S1: Photo-image of 0.1 M pyrene dicarboxylic acid solution in acetonitrile containing 5% NaOH aqueous solution (a), schematic of experimental procedure (b), actual images from first (c), middle (d) and after 150 min (e) of BPE operation. The proposed chemical structure (f).

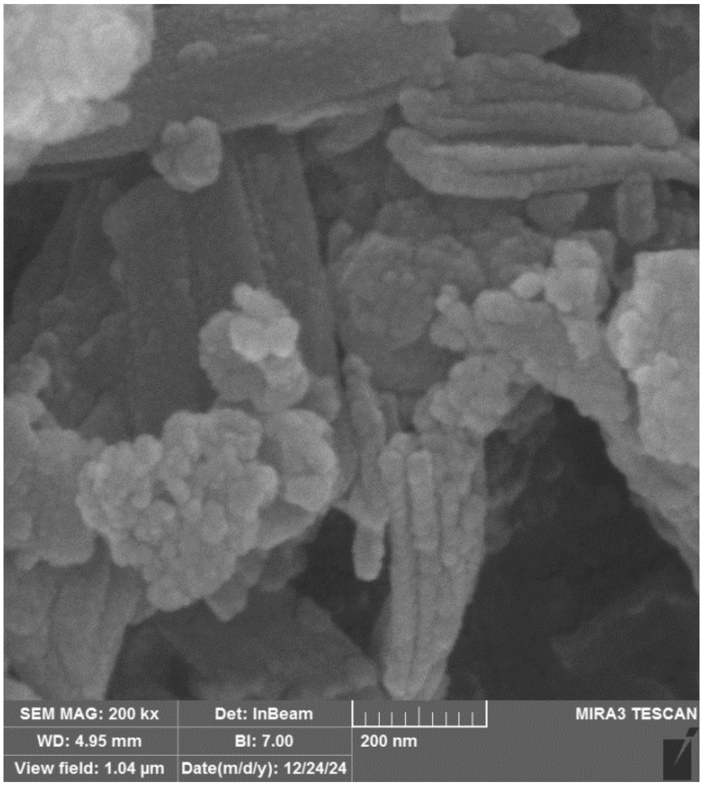

Figure S2: Higher magnification SEM image of resulted Cu-py MOF (the scale bar is 200 nm).

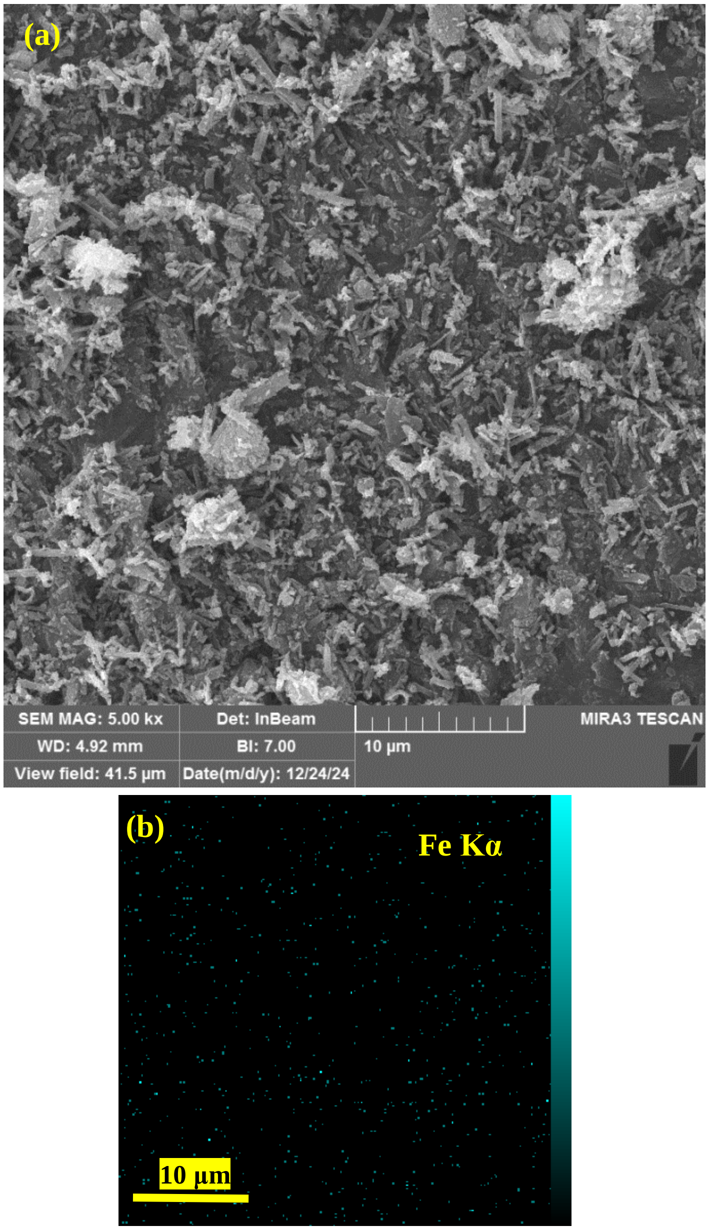

Figure S3: Wide area SEM image with scale bar of 10 μm, scanned for taking of map image (a) and corresponding map image of Fe element with the same scale bar (b).

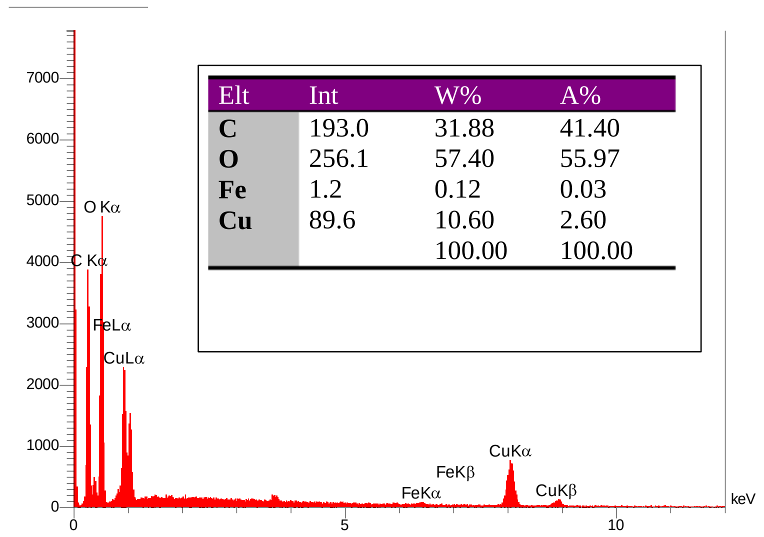

Elt	Int	W%	A%
**C**	193.0	31.88	41.40
**O**	256.1	57.40	55.97
**Fe**	1.2	0.12	0.03
**Cu**	89.6	10.60	2.60
		100.00	100.00

Figure S4: Energy dispersive X-ray (EDX) analysis of resulted Cu-py MOF. The inset displays the corresponding elemental percentages in the structure.

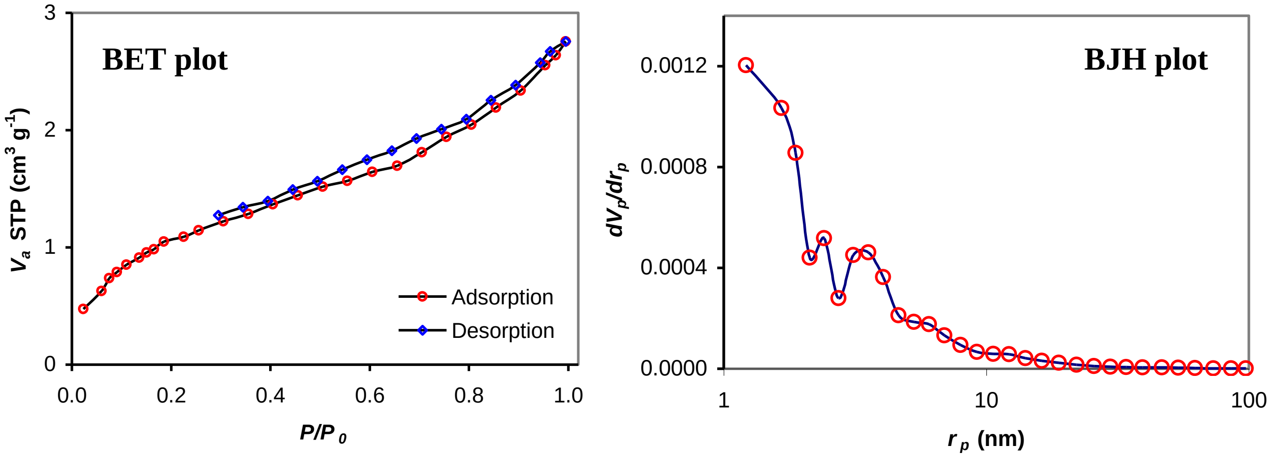

Figure S5: BET and BJH plots of resulted Cu-py MOF.

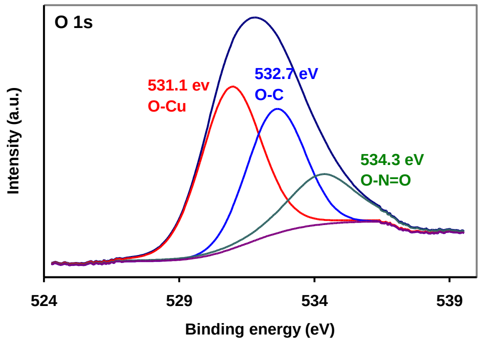

Figure S6: Fitting of XPS narrow scan for O 1s.

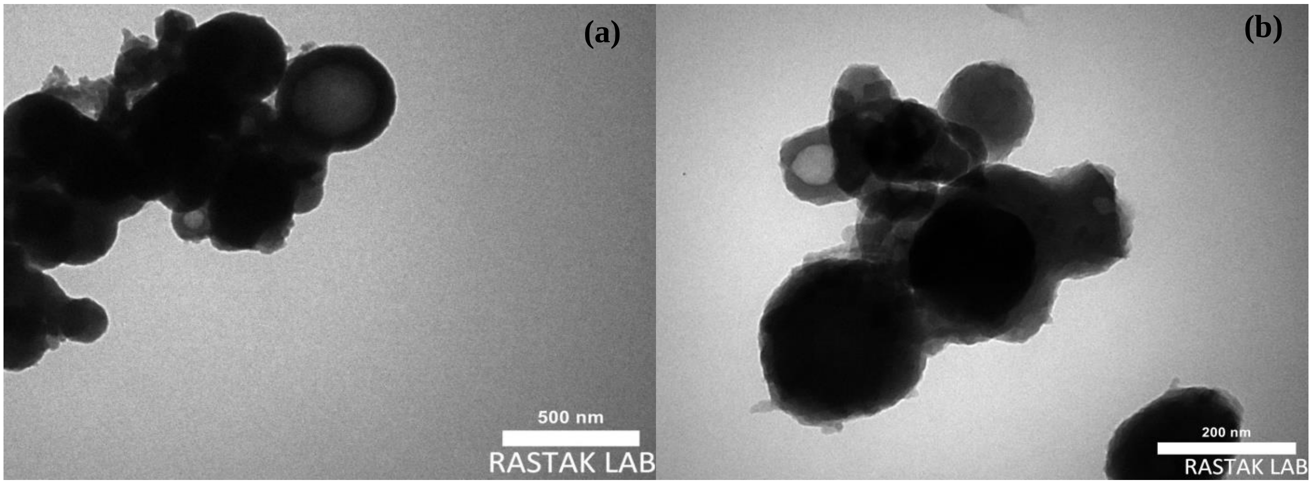

Figure S7: TEM images of resulted Cu-py MOF with different magnifications: a) scale bar is 500 nm and b) scale bar is 200 nm.

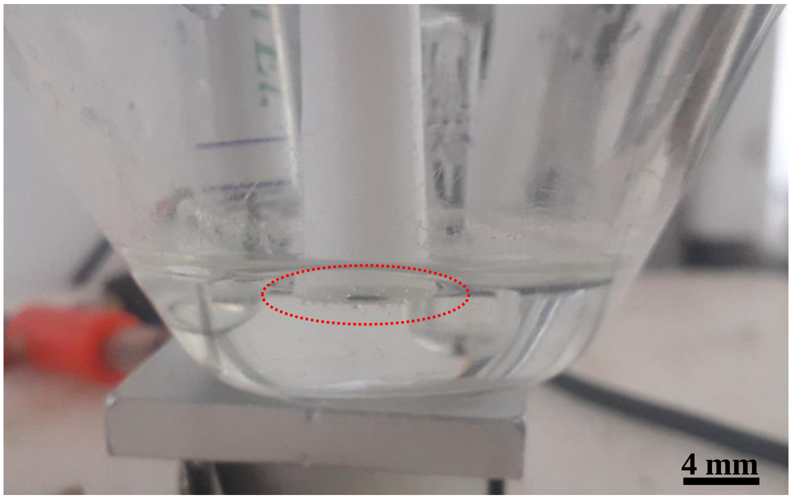

Figure S8: Actual photo-image of electrochemical cell after light illumination for one hour at input potential of -0.3 V vs. SCE. As can be seen, so many bubbles have gathered on the Teflon cover of glassy carbon electrode (the scale bar is 4 mm).

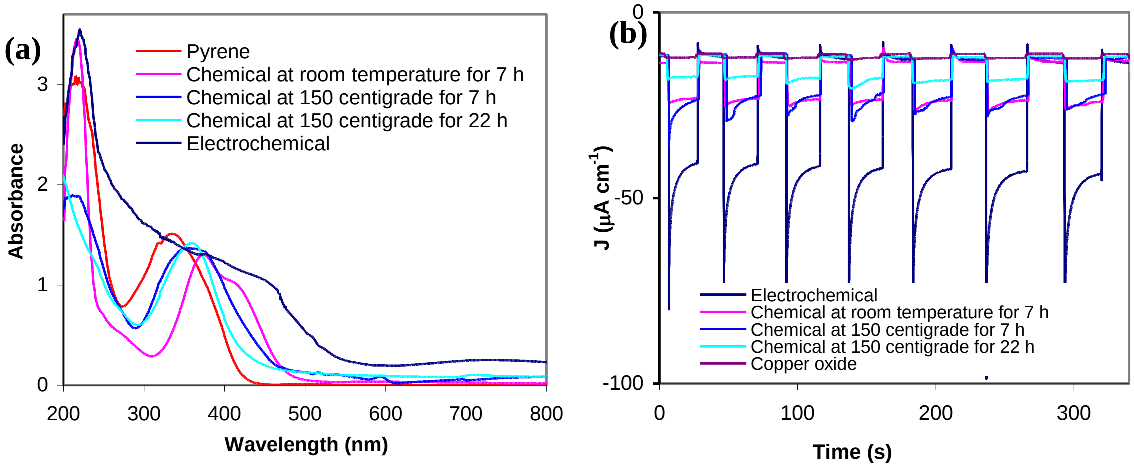

Figure S9: Uv-Vis spectra for Pyrene and different Cu-py synthesized through different electrochemical (BPE), room temperature and solvothermal conditions (a) and corresponding photocatalytic activities of at potential of 0.3 V vs. RHE.

METHOD S2

Calculation of released amount of H_2 during photocatalysis experiment:

$$n\ (mole) = \frac{Q(colon)}{F(colon\ per\ mole)} \qquad \text{(S1)}$$

where n is the mole number of transferred e^-, Q=i×t represents the generated colons during photocatalysis, F is the faradaic number equal to 96485 colons per mole. When for each mole H_2, 2 mole e^- are required, so the amount of released H_2 equals to 0.5 mole number of electron. Based on the gas equation relationship, the volume of evaporated H_2 can be calculated as follow:

$$PV=nRT \qquad \text{(S2)}$$

where P=0.8 atmosphere, the pressure of laboratory, R=0.083 lit.atm.K^{-1}.mol^{-1} is the general gas constant and T=296 K is the temperature of laboratory. So, the amount of released H_2 per one cm^{-2} of catalyst at one hour is obtained as much as 30.1 μL.

REFERENCES

[1] Frisch, M. J.; Trucks, G. W.; Schlegel, H. B.; Scuseria, G. E.; Robb, M. A.; Cheeseman, J. R.; Zakrzewski, V. G.; Montgomery, J. A.; Stratmann, JrR. E.; Burant, J. C.; Dapprich, S.; Millam, J. M.; Daniels, A. D.; Kudin, K. N.; Strain, M. C.; Farkas, O.; Tomasi, J.; Barone, J.; Cossi, M.; Cammi, R.; Mennucci, B.; Pomelli, C.; damo, C. A.; lifford, S. C.; chterski, J. O.; Petersson, G. A.; Ayala, P. Y.; Cui, Q.; Morokuma, K.; Malick, D. K.; Rabuck, A. D.; Raghavachari, K.; Foresman, J. B.; Cioslowski, J.; Ortiz, J. V.; Baboul, A. G.; Stefanov, B. B.; Liu, G.; Liashenko, A.; Piskorz, P.; Komaromi, I.; Gomperts, R.; Martin, R. L.; Fox, D. J.; Keith, T.; Al-Laham, M. A.; Peng, C. Y.; Nanayakkara, A.; Gonzalez, C.; Challacombe, M.; Gill, P. M. W.; Johnson, B.; Chen, W.; Wong, M. W.; Andres, J. L.; Gonzalez, C.; Head-Gordon, M.; Replogle, E. S.; Pople, J. A. Gaussian 98, revision A.7; Gaussian, Inc: Pittsburgh PA, 1998.

[2] Tomasi J, Persico M (1994) Chem Rev 94:2027.

[3] O'Boyle NM, Tenderholt AL, Langner KM, Cclib: A library for package independent computational chemistry algorithms, J Comput Chem 29:839–845, 2008.
